# UNDERSTANDING THE RELATIONSHIP BETWEEN AIR QUALITY AND FIVE-YEAR SURVIVAL OF PATIENTS IN THE AMERICAN FAMILY COHORT

**DOI:** 10.1093/geroni/igad104.3794

**Published:** 2023-12-21

**Authors:** Divya Periyakoil, Ndola Prata, Marie Diener-West

**Affiliations:** Johns Hopkins Bloomberg School of Public Health, Baltimore, Maryland, United States; University of California, Berkeley, Berkeley, California, United States; Johns Hopkins, Baltimore, Maryland, United States

## Abstract

Air pollution is an important social determinant of health and associated with cardiopulmonary disease, cancer, diabetes, and cognitive impairment. This study examined the impact of air pollution on the mortality of patients in the American Family Cohort (AFC), a national dataset of 6.6 million Americans, derived from the American Board of Family Medicine PRIME Registry electronic health record data. The AFC data were merged with the Environmental Protection Agency’s Air Quality Index (AQI) measures for the study period (2016-2022). Two Cox-Regression-Models were performed to determine how (1) baseline air quality and (2) air quality measures over time would affect patients’ five-year survival. Fixed covariates of interest--gender, race, age, location, and year of entry into the study--were studied as well. The adjusted hazard of death in the group with baseline AQI greater than 50 was 4.02 times higher than that of the group with AQI less than or equal to 50 (95% CI: 3.36, 4.82, p < 0.05). Compared to younger patients, the hazard of death was 6.73 times higher in persons older than 80 years of age (95% CI: 5.47, 8.28; p < 0.05). Black/African American patients had a 4.27 times higher hazard of death (95%CI: 3.47, 5.26; p < 0.05) compared to other races. Our study shows that vulnerable populations (older adults and persons from minority communities) are disproportionately burdened by poor air quality. Public health initiatives that focus on improving air quality for minority communities will improve their longevity.

